# Effect of maternal smoking during pregnancy on child blood pressure in a European cohort

**DOI:** 10.1038/s41598-022-21337-7

**Published:** 2022-10-15

**Authors:** Ester Parada-Ricart, Veronica Luque, Marta Zaragoza, Natalia Ferre, Ricardo Closa-Monasterolo, Berthold Koletzko, Veit Grote, Dariusz Gruszfeld, Elvira Verduci, Annick Xhonneux, Joaquin Escribano

**Affiliations:** 1 Paediatric Nutrition and Human Development Research Unit, Universitat Rovira i Virgili, IISPV, Sant Llorenç 21, 43201 Reus, Spain; 2 Department of Pediatrics, Hospital Universitari de Tarragona Joan XXIII, Dr. Mallafré i Guasch 5, 43007 Tarragona, Spain; 3 Hospital Universitari Sant Joan de Reus, 43204 Reus, Spain; 4 Division of Metabolic and Nutritional Medicine, Department of Pediatrics, Dr. Von Hauner Children’s Hospital, LMU University Hospital Munich, Lindwurmstr. 4, 80337 Munich, Germany; 5 Neonatal Department, Children’s Memorial Health Institute, 04-730 Warsaw, Poland; 6 Department of Pediatrics, V. Buzzi Children’s Hospital, Milan, Italy; 7 Department of Health Sciences, University of Milan, Milan, Italy; 8 Clinique CHC MontLegia, Liège, Belgium

**Keywords:** Health care, Risk factors, Cardiovascular diseases

## Abstract

Hypertension is a public health issue that can have its origin in the early phases of development. Maternal smoking during pregnancy (MSDP) could play a role in offspring’s cardio-metabolic programming. To assess the relationship between MSDP and later blood pressure (BP) in children we conducted a secondary analysis of a randomized dietary intervention trial (EU-Childhood Obesity Project). Healthy term infants with normal birth weight were recruited during the first 8 weeks of life in 5 European countries and followed until 11 years of age. Data on MSDP was collected at recruitment. BP and anthropometry were assessed at 11 years of age. Children were classified according to AAP guidelines as normal BP: BP < 90th percentile; high BP: ≥ 90th percentile with the subset of children having BP > 95th percentile categorized as hypertensive. Out of 572 children, 20% were exposed to MSDP. At 11 years, 26.8% had BP over the 90th centile. MSDP beyond 12 weeks of gestation was associated with higher systolic BP percentile (adjusted B 6.935; 95% CI 0.454, 13.429; p = 0.036) and over twofold increase likelihood of hypertension (OR 2.195; 95% CI 1.089, 4.423; p = 0.028) in children at 11 years. MSDP was significantly associated with later BP in children.

## Introduction

Hypertension is much more common among children and adolescents than previously thought^1^ and frequently translates into adult hypertension^2^. Hypertension increases the risk of heart disease and stroke which are leading causes of death in adulthood in developed countries^3^.

Several factors have been linked to an increase in the incidence of hypertension in children and adults: obesity, low physical activity, high intake of processed food, high consumption of sodium and environmental contamination^4–7^. The deleterious effect of cigarette smoking on cardiovascular diseases in adults is well documented^8^.

The Developmental Origins of Health and Disease hypothesis, which developed from studies identifying links between birth weight and adult cardiovascular disease mortality, suggests cardiometabolic diseases have their origins in early life^9^. This hypothesis hints that early-life factors, including genetic, developmental and environmental factors, could permanently affect body structure and function, and contribute to diseases later in life. Epigenetic mechanisms defined as heritable changes other than those in the DNA sequence (DNA methylation and post-translational modification of histones)^10^ are believed to account for some of the observations based on the Developmental Origins of Health and Disease approach.

Maternal smoking during pregnancy (MSDP) is known to be associated with adverse birth outcomes (stillbirth, neonatal death, and perinatal death)^11^ as well as low birth weight^12^. Several studies have linked MSDP to long-term consequences on children’s health^13^ especially on increasing obesity risk^14–16^. There is also growing evidence that MSDP may increase the risk of diabetes and metabolic syndrome^17,18^ as well as contribute to the development of hypertension. These effects are suggested to be associated with differential methylation of cytosine–phosphate–guanine base pairs in the newborn^19^ that can be persistent up to the adolescence when exposed early to tobacco^20,21^.

Large evidence reveals an inverse association between birth size and blood pressure (BP) in children and adults, however, birth size may just be a marker of other genetic and environmental factors that are the true etiologic factors for hypertension. Controversial results exist regarding the effect of MSDP on BP: some studies reveal that heavy MSDP is significantly associated with higher systolic BP, independently of intrauterine growth restriction but probably mediated by childhood body mass index (BMI) or weight trajectory^22^, while in other cohorts this association has not been identified^23,24^.

We aim to assess if there is a relationship between MSDP and the development of elevated BP/ hypertension later in life in a European cohort of children born at term with normal birth weight; furthermore, we aim to analyze whether the protein content in baby-formula could be a contributor that modulates this relationship.

## Methods

This is a secondary analysis of a randomized dietary intervention trial EU-Childhood Obesity Project (EU-CHOP) (NCT00338689)^25^ at 11 years of follow-up.

Briefly, healthy singleton term infants with a normal weight for gestational age (birth weight ≥ 2500 gr) who were born between October 2002 and July 2004 were recruited in 5 countries (Belgium, Germany, Italy, Poland, and Spain) during the first 8 weeks of life. Formula-fed infants were randomly assigned to either a lower (1.25 g/dL and 2.05 g/dL of protein in the infant formula and follow-on formula) or a higher protein content formula (1.6 g/dL in infant formula and 3.2 g/dL in follow-on formula, respectively) to assess the effect of protein supply during the first year of life on later obesity risk. A group of breastfed (for at least 3 months) infants was recruited and followed up as the observational gold standard group. Further details on the clinical trial are published elsewhere^25,26^.

Information on the course of pregnancy, medical history, parent’s pre-pregnancy weight, and height, and child anthropometric data at birth was obtained from standardized parent interviews at the recruitment visit (first child’s visit).

Information regarding parents’ highest educational level was collected from the participants at the time of recruitment and recorded according to the International Standard Classification of Education—ISCED-97^27^, and classified as low (pre-preliminary and primary level of education), medium (secondary and post-secondary level of education) and high (tertiary level of education).

Self-reported data about maternal smoking (and amount of cigarettes/day if smoking) in the 3 months before pregnancy, smoking up to the day pregnancy confirmed, during early pregnancy (< 12th week) and beyond the 12th week, as well as maternal alcohol consumption during pregnancy was retrospectively gathered by means of questionnaire at the recruitment visit. We categorized mothers into three groups: no smoking during gestation, smoking just up to 12th week of gestation and smoking beyond 12th week of gestation.

Anthropometry was measured at the 11 years visit. Weight was measured in underwear on a SECA 702/703 digital scale (10 g precision) and height was measured with a SECA 242 digital stadiometer (1 mm precision). Ponderal index (kg/m^3^) was calculated at birth using the formula weight (kg)/length^3^ (m^3^) and BMI (kg/m^2^) was calculated at each encounter using the formula weight (kg)/height^2^ (m^2^).

At 11 years, BP (mmHg) was measured using a Dinamap ProCare 100/200 digital blood pressure monitor following a standardized procedure. BP was measured after at least 15 min from arrival at the center and after at least 5 min of rest. The measurement was taken in duplicate and on the left arm supported by a slightly elevated horizontal support (that is, close to the level of the heart). Both measurements were taken separated by at least 5 min, and the mean between them was used for statistical analysis.

### Data analysis

Continuous variables are shown as mean and standard deviation or as median and interquartile range according to their distribution. The distribution of the variables was assessed through graphical representation. Categorical variables are described as N and percentage of the total.

Weight, height, and BMI z-scores were calculated using World Health Organization (WHO) references^28^. Values of BP were standardized as percentiles according sex, age and height using the references from the American Academy of Pediatrics and categorized according to its definition as normal, elevated BP (≥ 90th percentile to < 95th percentile or 120/80 mmHg to < 95th percentile whichever is lower), stage 1 hypertension (≥ 95th percentile to < 95th percentile + 12 mmHg, or 130/80 to 139/89 mmHg, whichever is lower) and stage 2 hypertension (≥ 95th percentile + 12 mmHg, or ≥ 140/90 mmHg, whichever is lower)^29^. We further classified patients as normal blood pressure (BP < 90th percentile and < 120/80) or high blood pressure (BP ≥ 90th percentile or ≥ 120/80).

The total number of cigarettes during gestation was obtained by adding the number of cigarettes smoked during each period:

- Until the gestation was confirmed: number of cigarettes/day × 7 days × number of weeks (gestational age when pregnancy was confirmed)
- Early pregnancy: number of cigarettes/day × 7 days × (12 − number of weeks when gestation was confirmed)
- Late pregnancy: amount of cigarettes/day × 7 days × (gestational age at delivery − 12).

Student T-test was used for 2-group comparisons and ANOVA for multiple group comparisons. Proportions were compared using Chi-square tests. The association between MSDP and children’s BP (SBP and DBP percentiles) at 11 years of age was evaluated by multiple regression analysis. We investigated whether the conclusions would be impacted by country heterogeneity by using mixed effect model analysis nested by country. The OR of having high blood pressure or hypertension was calculated using binomial logistic regression. Based on previous literature, we analysed the effect of potential confounders such as country, sex, socioeconomic status (assessed as mothers’ highest educational level), pregestational maternal BMI, paternal BMI, ponderal index at birth, and child’s BMI z-score at 11 years of age. We also analysed the effect of breastfeeding or feeding with different protein-contain formula on blood pressure at 11 years.

We assessed the effect of maternal smoking:

- Considering MSDP as a 2-categories variable: no/yes MSDP,
- Considering each period individually: prior pregnancy, first 12 weeks of gestation and beyond 12 weeks of gestation.

Among the mothers who smoked during pregnancy, we assessed the association between the number of cigarettes and the systolic and diastolic blood pressure percentile at 11 years.

Missing data: Regression analysis were performed including just complete cases.

Statistical significance was accepted at the level of p < 0.05.

Statistical analyses have been conducted with IBM SPSS Statistics for Windows, Version 27.0 Released 2020 (IBM Corp. Armonk, NY: IBM Corp). We used RStudio 2021.09.0 to perform mixed model analysis (package lme4).

The datasets analysed during the current study will be available from the corresponding author on reasonable request.

### Ethics

The project was accepted by the correspondent Ethics committees responsible in each study centre: Ethik-Kommission der Bayerishen Landersäzrtekammer from Munich, Comité d’Ethique d’Hopital Universitarie des Enfants Reine Fabiola from Brussels, Comité d’Ethique du Clinique Saint Vincent from Liege, Comitato Etico Azienda Ospedaliera San Paolo Polo Universitario from Milano, Komiscja Bioetyczna Pomnik-Centrum Zdrowia Dziecka from Warsaw, Comitè d’Ètica d’Investigació Clínica de l’Hospital Universitari Sant Joan de Reus i Comitè d’Ètica d’Investigació Clínica de l’Hospital Universitari de Tarragona Joan XXIII. Written informed consent was obtained from parents or caregivers. All research was performed following the Declaration of Helsinki.

## Results

Out of 1678 children recruited in the original study, 572 patients had data regarding MSDP and BP at 11 years. Sociodemographic data at recruitment, smoking status of the mother prior and during pregnancy (first trimester and beyond), anthropometric data from parents and child at birth, and feeding type during the first year as well as BP and anthropometry at 11 years of age are displayed in Table 1. There were no significant differences in sex, father’s and mother’s BMI before pregnancy, ponderal index at birth and feeding between patients lost to follow-up at 11 years and included patients. Mothers of children with complete follow-up had significantly higher educational level and smoked less frequently during pregnancy than mothers of children lost to follow up (15.4% vs. 24.8%, respectively) (supplementary file. Table S1).

**Table 1:** Sociodemographic data, smoking status of the mother prior and during pregnancy (first trimester and beyond), anthropometric data from parents and children (at birth and at 11 years of age) and blood pressure at 11 years.

	N (%) /mean (SD)
Total	572
**Data at recruitment**	
Country	
Spain	177 (30.9%)
Italy	145 (25.3%)
Poland	94 (16.4%)
Germany	80 (14.0%)
Belgium	76 (13.3%)
Mother’s education level^a^	
No/low	95 (16.6%)
Middle	293 (51.2%)
High	182 (31.8%)
Single mother (yes)	22 (3.9%)
Mother's age at child's birth (years)	31.3 (4.6)
Mother's BMI before pregnancy (kg/m^2^)	23.4 (4.2)
Father's BMI before pregnancy (kg/m^2^)	26.2 (3.6)
Alcohol during pregnancy (yes)	129 (22.6%)
Mother smoking	
Prior pregnancy	176 (30.8%)
Up to pregnancy confirmation	154 (26.9%)
Up to 12th week	108 (18.9%)
Beyond 12th week	88 (15.4%)
Any during pregnancy	114 (19.8%)
Number of cigarettes^b^	1078 (1988)
Sex (male)	274 (47.9%)
Gestational age (weeks)	39.8 (1.2)
Caesarean section (yes)	130 (22.8%)
Birth weight (kg)	3.28 (0.34)
Birth length (cm)	50.6 (2.5)
Ponderal index at birth (kg/m^3^)	2.55 (0.29)
Feeding type	
Formula fed	378 (66.2%)
Low protein	193 (33.8%)
High protein	185 (32.3%)
Breastfed	193 (33.8%)
**Data at 11 years of age**	
Child BMI category (WHO 2007)	
Underweight	13 (2.3%)
Normal	370 (64.7%)
Overweight	124 (21.7%)
Obese	64 (11.2%)
Height (cm)	147 (126–171)
Systolic blood pressure (mmHg)	108 (83–144)
Diastolic blood pressure (mmHg)	59 (36–85)
Blood pressure (category)^c^	
Normal	419 (73.2%)
Elevated blood pressure	57 (10.0%)
Stage 1 hypertension	84 (14.7%)
Stage 2 hypertension	12 (2.1%)

^a^Mother’s education level: low (pre-preliminary and primary level of education), medium (secondary and post-secondary level of education) and high (tertiary level of education).

^b^Total number of cigarettes smoked during pregnancy considering just mothers who smoked during pregnancy: median (interquartile range).

^c^Blood pressure category based on AAP definitions: normal: blood pressure < 90th percentile and < 120/80; elevated blood pressure: ≥ 90th percentile to < 95th percentile or 120/80 mmHg to < 95th percentile whichever is lower; stage 1 hypertension: ≥ 95th percentile to < 95th percentile + 12 mmHg, or 130/80 to 139/89 mmHg, whichever is lower; Stage 2 hypertension: ≥ 95th percentile + 12 mmHg, or ≥ 140/90 mmHg, whichever is lower.

Maternal smoking prevalence was quite different between countries, ranging from 20% in Belgium to 42% in Poland before gestation, and from 11% in Germany to 24% in Spain after 12 weeks of gestation. Twenty-eight mothers reported to smoke ≥ 10 cigarettes per day during late pregnancy.

At 11 years, 26.8% of the children had high BP.

Parents and child characteristics of children with normal BP vs. children with high BP (BP ≥ 90th percentile or ≥ 120/80 mmHg) are displayed in Table 2.

**Table 2:** Mother and child characteristics according to children blood pressure status at 11 years.

	Normal BP N = 418		High BP^a^ N = 153		p-value
	N	Mean (SD)/%	N	Mean (SD)/ %	
**Prenatal and social factors**					
Mother's age at child's birth	419	31.34 (4.53)	153	31.26 (4.91)	0.845
Mother's BMI before pregnancy	403	23.09 (3.99)	149	24.29 (4.52)	**0.003**
Father's BMI before pregnancy	417	25.96(3.60)	150	26.88 (3.61)	**0.007**
Single mother (yes)	9	2.2%	13	8.5%	**< 0.001**
Mother’s education level					
Low	67	16.1%	28	18.3%	0.372
Middle	210	50.4%	83	54.2%	
High	140	33.6%	42	27.5%	
**Toxics during gestation**					
Alcohol during pregnancy (yes)	87	21.3%	42	27.6%	0.115
Smoked prior pregnancy (yes)	119	28.5%	57	37.3%	**0.044**
Smoked early pregnancy (< 12th week) (yes)	68	16.2%	40	26.1%	**0.007**
Smoked beyond (beyond 12th week) (yes)	54	12.9%	34	22.2%	**0.007**
Any smoking during pregnancy (yes)	72	17.2%	42	27.5%	**0.007**
Total cig smoke during the whole pregnancy	418	330 (838)	148	568 (1142)	**0.020**
**Children data**					
Gestational age (weeks)	424	39.8 (1.21)	149	39.7 (1.22)	0.092
Caesarean section (yes)	90	21.6%	40	26.3%	0.234
Sex (female)	215	51.3%	83	54.2%	0.534
Birth weight (kg)	419	3.29 (0.35)	153	3.29 (0.33)	0.867
Birth length (cm)	417	50.61(2.56)	153	50.64 (2.49)	0.905
Ponderal index at birth (kg/m^3^)	417	2.55 (0.29)	153	2.55 (0.31)	0.937
Child was formula-fed (yes)	274	65.4%	104	68.0%	0.564
Low protein	138	33.0%	55	35.9%	
High protein	136	32.5%	49	32.0%	
BMI z-score at 11 years	418	0.22 (1.20)	153	0.95 (1.17)	**< 0.001**
Height (cm)	419	146.9 (7.1)	153	149.3 (6.8)	**< 0.001**
Waist circumference at 11 years	419	65.75 (9.14)	153	70.98 (9.57)	**< 0.001**

Significant values are in bold.

^a^High blood pressure defined as ≥ 90th percentile or ≥ 120/80 mmHg whatever is lower according AAP guidelines.

Mother’s age, mother’s educational level and alcohol during pregnancy as well gestational age, sex, breastfeeding and anthropometry at birth were similar in both groups. Parents of children in the group with high BP had a significantly higher BMI before pregnancy. BMI and waist circumference at 11 years was higher in the children with high BP than in children with normal BP. Maternal smoking was also different in the two groups.

### Prediction of blood pressure percentile at 11 years of age

MSDP beyond 12th week of gestation was positively associated with SBP percentile at 11 years of age in models adjusted by sex, country, mother’s educational level and father and mother’s BMI before pregnancy (Table 3 model 1). Further adjustment by feeding, ponderal index at birth and BMI z-score at 11 years, which could be mediators of the effect of MSDP on blood pressure, improved the model and the effect of MSDP remained significant (Table 3 model 2). BMI has a greater association than in utero smoking exposure to SBP percentile at 11 years of age.

**Table 3:** Lineal regression models assessing the effect of maternal smoking beyond 12th week of gestation on SBP percentile at 11 years.

	B (95% CI) (unit in percentile)	p- value	Adjusted R^2^
**Model 1**			
Smoked beyond 12th week (yes)	7.862 (1.155, 14.570)	**0.022**	0.138
Mother's education level	− 0.068 (− 3.732, 3.597)	0.971	
Mother's BMI	0.896 (0.316, 1.476)	**0.003**	
Father's BMI	0.916 (0.264, 1.568)	**0.006**	
**Model 2**			
Smoked beyond 12th week (yes)	6.942 (0.454, 13.429)	**0.036**	0.243
Mother's education level	− 0.133 (− 3.644, 3.377)	0.940	
Mother's BMI	0.184 (− 0.384, 0.752)	0.524	
Father's BMI	0.237 (− 0.393, 0.866)	0.461	
Ponderal index at birth	2.711 (− 7.241, 12.663)	0.593	
Breastfeeding	− 1.052 (− 6.543, 4.438)	0.707	
Low protein formula feeding	− 3.395 (− 8.808, 2.019)	0.219	
BMI z-score at 11 years	8,352 (6.394, 10.311)	**< 0.001**	

Sample size 542 patients because of missing data.

Model 1 adjusted by country and sex.

Model 2 adjusted by country, sex, and other post-exposure potential confounders such as child’s anthropometry or feeding (comparison group for feeding high protein).

Significant values are in bold.

Breast-feeding did not predict SBP percentile in the fully adjusted model that compared breast-feeding vs formula feeding (coefficient for breastfeeding − 0.574, 95% CI − 5.41, 4.31; p = 0.817).

Performing mixed effect model analysis nested by country did not change the results (supplementary file: Table S3).

We did not identify a significant effect of MSDP on DBP at 11 years of age (supplementary file Table S3).

Smoking beyond 12th week of gestation significantly increased the likelihood of high blood pressure (OR 1.9, 95% CI 1.027, 3.514; p = 0.041) and the likelihood of hypertension (stage 1 or 2) at 11 years (OR 2.195 (95% CI 1.089, 4.423; p = 0.028). Smoking before pregnancy did not significantly increase the likelihood of high blood pressure (OR 1.61, 95% CI 0.980, 2.666, p = 0.060) or hypertension at 11 years (OR 1.473, 95% CI 0.815, 2.662, p = 0.200).

The effect of MSDP on SBP percentile differed when we considered smoking in different stages of gestation. In the regression analysis, the coefficients for MSDP in different stages of gestation for predicting SBP percentile at 11 years are shown in Table 4. The effect of MSDP did not reach significance when we considered MSDP just as 2 categories (“any smoke during pregnancy”) or when we considered smoking during the first 12 weeks of gestation (yes/no) as a predictor. When we included in the model “smoking beyond 12th week” (yes/no), it showed a significant effect on SBP percentile.

**Table 4:** Effect of maternal smoking on offspring systolic blood pressure percentile at 11 years by assessing maternal smoking in different stages of gestation.

	N	B^a^	95% CI		p-value	R^2^ ajust
			Inf	Sup		
Smoked prior pregnancy	176	4.194	− 0.909	9.297	0.107	24.1
Smoked during early pregnancy	108	4.462	− 1.507	10.431	0.143	24.1
Smoked beyond 12th week	88	6.935	0.452	13.419	**0.036**	24.4
Any smoke during pregnancy	114	5.573	− 0.334	11.481	0.064	24.2

Sample size 542 patients because of missing data.

Significant values are in bold.

^a^Coefficient in the linear regression model to predict SBP percentile at 11 years of age (percentile unit) adjusted by country, mother’s educational level, mother, and father’s BMI before pregnancy, feeding type, ponderal index at birth, and BMI z-score at 11 years.

Among the 114 mothers who smoked during pregnancy, we had data on the average cigarette/day in 113. Number of cigarettes smoked during gestation ranged from 28 to 5880, median 1078 cigarettes. Number of cigarettes/day among mother who smoked after 12th week of gestation ranged from 1 to 20, median 5 cigarettes. Neither the number of cigarettes, nor the cigarettes/day during the late pregnancy predicted SBP percentile at 11 years in our population (supplementary file: Tables S4 and S5).

## Discussion

In our cohort of children born in the early XXI century at term and with normal birth weight, maternal smoking beyond 12th week of pregnancy had a significant effect on SBP at 11 y ears of age. The effect of MSDP on BP/hypertension had been studied in other cohorts with different results (Table 5). The age at which BP/hypertension was assessed (from 4 weeks of age to adulthood), the period when children were born (from 1958 to 2006) with the subsequent differences in pregnancy and postnatal care, and the different geographic settings (12 in European countries, 2 in the USA, 2 in South America, 3 in Australia/New Zeeland, and 1 in Israel) could explain the differences. Also, adjustment for possible confounding factors, which was very different between the studies, could be responsible for the discrepancy in the results.

**Table 5:** Summary of studies on the effect of maternal smoking during pregnancy on offspring blood pressure.

Primary studies		
Country/birth (cohort)	Age	Result
Netherlands/2001–2002 (UHP)^30^	4 weeks	New-borns of smoking mothers had a 5.4 mmHg higher SBP than offspring of mothers who were not exposed to tobacco smoke in pregnancy, no differences in DBP
USA/1999–2002 (Project Viva)^31^	3 y	MSDP was not associated with SBP
UK/1984–5^32^	4 y	No difference in BP due to MSDP
Portugal/2005–2006 (Generation XXI)^33^	4 y	Offspring of MSDP presented a higher SBP z-score (β = 0.08, 95% CI 0.04 to 0.14. No effect on DBP
Australia/1981–4 (MUSP)^34^	5 y	SBP of children exposed to MSDP was on average 0.92 mmHg (95% CI 0.17 to 1.68) greater than that of children whose mothers had never smoked
Sweden/2006–2011 (Snus study cohort)^35^	5–6 y	Children prenatally exposed to snus had a mean SBP of 99 (SD, 7.1) mmHg, which was higher than in control children (93 [SD, 5.8] mmHg; P = 0.013). No differences in DBP
Australia/1989 (Raine Study)^36^	6 y	Children from MSDP had a higher SBP (1.20 mmHg (95% CI 0.00–2.30). This observed association was dependent on birth weight
UK/1981 (preterm < 1850 g)^37^	8 y	The group born < 33 weeks gestational age from smoking mothers had lower SBP and DBP than those with non-smoking, whereas at 33 or more, the relationship was reversed (rise of 2.9 mmHg in SBP for every 10 cigarettes smoked (95% CI 1.0, 4.8)
Argentina/1995–6^38^	5–9 y	No association of MSDP with SBP in the adjusted analysis
UK/1991–2 (ALSPAC)^39^	7 y	MSDP was associated with SBP (β = 0.86 95% CI 0.26–1.46) p = 0.005, not with DPB (not adjusted for confounding factors)
UK/1991–2 (ALSPAC)^40^	7 y	In age- and sex-adjusted models MSDP modest increases SBP, adjusting for all the confounders attenuated this association (0.05, IC95% -0.59, 0.68)
USA/1959–65 (CPP cohort)^22^	7 y	Heavy MSDP was significantly associated with higher offspring SBP (adjusted mean difference versus non-smoking: 0.73 mmHg [95% CI 0.32,1.14], which attenuated to null (0.13 [95% CI − 0.27, 0.54]) after adjustment for changes in BMI
New Zeland/1972–3 (Dunedin study)^41^	9 y	Being a twin, MSDP, BMI, and birth weight were significantly associated with BP, adjusted for the other variables β = 1.34 (IC95% 0.31,2.38)
Europe/2003–2009 (HELIX)^23^	6–11 y	No significant effect of MSDP on BP
**Blood pressure assessed during adulthood**		
Israel/1974–1976 Jerusalem cohort^42^	17–32 y	MSDP had no significant effect on BP after adjustment for potential confounding factors
Sweden/1983–1988^43^	18 y	MSDP of at least 10 cigarettes daily (vs. non-smoking) exhibited a small but significant on SBP (b = 0.53, 95% CI 0.26 to 0.80) and on DBP (b = 0.4495% CI 0.30–0.57)
Australia/1981–4 (MUSP)^44^	21 y	No differences on mean SBP or DBP (unadjusted)
UK/1958 (British Birth Cohort)^45^	Adults	At 45 years of age, MSDP was not associated with BP after adjustment for maternal, early life and adult covariates
Brazil/1982Pelotas cohort^46^	Adults	At 22–23 years of age, MSDP was not associated with BP
Denmark/born < 1990 (GESUS)^47^	Adults	At a mean age of 55.4 years (SD 14) MSDP was not a risk factor for HTA (OR = 1.13, 95% IC 0.92–1.38)

Meta-analysis		
Author	Main results	
Brion 2008^48^	10 studies MSDP was associated with a modest increase in offspring BP (0.67 mmHg unadjusted, 0.62 mmHg)	
Parmar 2018^18^	22 studies, 18,212 participants Lower DNA methylation at cg14179389, the strongest maternal prenatal smoking locus, was associated with increased WC and BP and Triglycerides when adjusted for sex, age and current smoking	

The observed prevalence of hypertension at 11 years in our study (16.8%) was higher than expected. We cannot exclude an overestimation of hypertension (we used a digital blood pressure monitor instead of auscultatory BP measurements defined in the current guidelines^29^) and also we did not confirm our values in 3 separate visits or by ambulatory blood pressure monitoring before diagnosing hypertension. But, since the prevalence of overweight/obesity in our cohort (32%) was also higher than expected, we think our results on BP may be the real picture of our children moving toward an increased cardiovascular risk phenotype as described in other studies: in the systematic review published by Song including 47 studies from different countries between 1990 and 2014, the prevalence of hypertension in children increased from 1.26% (95% CI 0.79–1.84%) in the 1990s to 3.30%; 95% CI 2.69–3.97% in the 2000s and up to 6.02% (95% CI 4.38–7.91%) in the period 2010 to 2014^1^.

The underlying mechanisms to explain the late effects of MSDP on cardiometabolic outcomes have been suggested to be due to epigenetic changes. The meta-analysis performed by Joubert et al. reported an association between sustained maternal smoking during pregnancy and differential DNA-methylation patterns in offspring cord blood. These epigenetic changes seem stable and independent of paternal smoking during the period of pregnancy, cumulative passive smoke exposure and adolescent smoking^21^. The translation of this differential methylation pattern on clinical outcomes is still under investigation^49^.

There is evidence on the influence of MSDP and other cardiovascular risk factors including increased adiposity and obesity in the offspring^44,47^. The complex interrelations of causality between prenatal/ postnatal environmental factors and vascular risk phenotype as well as the interactions between the different components of this phenotype (obesity, hypertension, insulin resistance, altered lipid profile, etc.) make it difficult to draw conclusions on the direct effect of MSDP on BP^24^.

Blood pressure is determined by the cardiac output and the peripheral resistance. Kidney plays a key role by acting on peripheral vascular resistance through the renin-angiotensin system and participates in the blood volume control by regulating urinary salt and water excretion. Autopsy studies have shown a strong correlation between renal weight, glomerular number, glomerular hypertrophy, and clinical signs of hypertension^50^. Several animal studies have assessed the effect of exposure to tobacco during gestation on the development of foetal kidney and later BP: In Balb/c mice, prenatal smoke exposure was associated with fewer nephron numbers at birth that persisted to adulthood; while glomerular volume was increased at 20 days of life but reduced in the adulthood^51^. In Wistar rats, prenatal exposure to waterpipe tobacco smoke during gestation negatively affected offspring birth weight, lowered the glomeruli area and increased significantly SBP in adult offspring rats^52^. In humans, continued maternal smoking during pregnancy is also associated with smaller combined kidney volume and lower eGFR in school-aged children^53^ so a direct effect of MSDP on BP cannot be excluded.

Since hypertension is a multifactorial disease, other aspects further in life (environmental, nutritional, behavioral, …) may change the relative influence of MSDP on BP. Our findings that the effect of MSDP on BP at 11 years of age was significantly influenced by BMI suggest that other contributors inducing a higher BMI or different body composition^54^ may play a major role in the development of high BP. In the study by Grijalva-Eternod, they demonstrated that height, relative lean mass and relative adiposity at 7–9 years of age were independently associated with greater BP.

We did not find a significant effect of infant feeding type (breastfeeding vs. formula feeding) on the prediction model for MSDP and blood pressure. This could be explained by the low number of children breastfed by mothers who smoked during gestation (12 out of 114). But also, the protein content in the formula fed infants did not predict blood pressure at 11 years of age in our population. Nevertheless, body composition was markedly influenced by infant feeding in this cohort^54^ and body composition is a known risk factor for metabolic syndrome. Since the relative effect of BMI on BP values looks to increase over time, we cannot rule out the influence of early nutrition factors in the development of hypertension later in life.

One important question is if there is a critical period of the gestation when maternal smoking is more deleterious. In our study, smoking during the first 12 weeks of gestation did not significantly predict SBP at 11 years as it did when we used as a predictor smoking beyond the 12th week of gestation.

This result can have different explanations: Previous studies have shown consistent linear dose-dependent associations of maternal smoking with foetal growth from early second trimester onwards with an increased risk of hypertension and metabolic syndrome in the adulthood in subjects with intrauterine growth restriction. The children included in our cohort had normal birth weight, but we cannot rule out there was some intrauterine growth restriction that could explain the association of maternal smoking during the second and third trimester to higher systolic blood pressure. Also, children from mother’s who didn’t quit smoking during first 12 weeks of pregnancy are exposed to more prolonged exposure with a possible cumulative damage. We did not find a significant association between total number of cigarettes smoked during pregnancy and blood pressure percentile at 11 years. So we should advise pregnant women to quit smoking as early as possible during pregnancy.We believe that tobacco exposure has a continuous and summatory effect on child’s health in agreement with the results of the meta-analysis performed by Joubert et al. that, despite the much larger number of women with any smoking during pregnancy (compared with sustained smoking), there were fewer statistically significant findings for this less specific exposure^19^.

A limitation of our study is the high attrition rate which could produce a selection bias of our population. No differences regarding sex, gestational age, birth weight, ponderal index at birth, father’s BMI before pregnancy, mother’s BMI before pregnancy and feeding between subjects with and without follow-up data at 11 years, but mother’s educational level and maternal smoking further 12th week of gestation was significantly different between patient with and without follow-up at 11 years.

Other limitations include that information on MSDP was self-reported retrospectively and we have no data on smoke exposure after birth (second-hand smoking) which could also have a relevant effect on blood pressure and on kidney function at 11 years of age. Another residual confounding can be also an issue (neighborhood-level socioeconomic status, maternal and paternal blood pressure, hypertensive disorders during gestation, diet, etc.). Also, we did not perform confirmatory measurements by auscultation or ambulatory blood pressure monitoring in subjects with SBP percentile > 95 to confirm hypertension as recommended in current guidelines.

Strengths of this study are the long follow-up, and the broad scope of the collected data.

In conclusion, the association of maternal smoking during pregnancy with blood pressure and hypertension suggests that maternal smoking during pregnancy may have a long-term effect on children’s BP. This information should be transmitted to pregnant women and healthcare workers to decrease the risk of adverse cardio-metabolic outcomes.

## Supplementary Information

Supplementary Tables.

## Data Availability

The datasets used and/or analyzed during the current study available from the corresponding author on reasonable request.

## References

[1] Song P (2019). Global prevalence of hypertension in children: A systematic review and meta-analysis. JAMA Pediatr..

[2] Chen X, Wang Y (2008). Tracking of blood pressure from childhood to adulthood: A systematic review and meta-regression analysis. Circulation.

[3] WHO|A global brief on hypertension. https://www.who.int/cardiovascular_diseases/publications/global_brief_hypertension/en/.

[4] Rosner B, Cook NR, Daniels S, Falkner B (2013). Childhood blood pressure trends and risk factors for high blood pressure: The NHANES experience 1988–2008. Hypertension.

[5] Maitre L (2018). Human Early Life Exposome (HELIX) study: A European population-based exposome cohort. BMJ Open.

[6] Malta D (2018). High sodium intake increases blood pressure and risk of kidney disease. From the Science of Salt: A regularly updated systematic review of salt and health outcomes (August 2016 to March 2017). J. Clin. Hypertens..

[7] Cheungpasitporn W (2015). Sugar and artificially sweetened soda consumption linked to hypertension: A systematic review and meta-analysis. Clin. Exp. Hypertens..

[8] Reitsma MB (2017). Smoking prevalence and attributable disease burden in 195 countries and territories, 1990–2015: A systematic analysis from the global burden of disease study 2015. Lancet.

[9] Barker DJP (2007). The origins of the developmental origins theory. J. Intern. Med..

[10] Callinan PA, Feinberg AP (2006). The emerging science of epigenomics. Hum. Mol. Genet..

[11] Pineles BL, Hsu S, Park E, Samet JM (2016). Systematic review and meta-analyses of perinatal death and maternal exposure to tobacco smoke during pregnancy. Am. J. Epidemiol..

[12] Kramer MS (1987). Determinants of low birth weight: Methodological assessment and meta-analysis. Bull. World Health Organ..

[13] Bell K (2018). The impact of pre and perinatal lifestyle factors on child long term health and social outcomes: A systematic review. Health Econ. Rev..

[14] Magalhães EIDS, Sousa BAD, Lima NP, Horta BL (2019). Maternal smoking during pregnancy and offspring body mass index and overweight: A systematic review and meta-analysis. Cad. Saude Publica.

[15] Philips EM (2020). Changes in parental smoking during pregnancy and risks of adverse birth outcomes and childhood overweight in Europe and North America: An individual participant data meta-analysis of 229,000 singleton births. PLoS Med..

[16] Vrijheid M (2020). Early-life environmental exposures and childhood obesity: An exposome-wide approach. Environ. Health Perspect..

[17] Behl M (2013). Evaluation of the association between maternal smoking, childhood obesity, and metabolic disorders: A national toxicology program workshop review. Environ. Health Perspect..

[18] Parmar P (2018). Association of maternal prenatal smoking GFI1-locus and cardio-metabolic phenotypes in 18,212 adults. EBioMedicine.

[19] Joubert BR (2016). DNA methylation in newborns and maternal smoking in pregnancy: Genome-wide consortium meta-analysis. Am. J. Hum. Genet..

[20] Banderali G (2015). Short and long term health effects of parental tobacco smoking during pregnancy and lactation: A descriptive review. J. Transl. Med..

[21] Rauschert S (2019). Maternal smoking during pregnancy induces persistent epigenetic changes into adolescence, independent of postnatal smoke exposure and is associated with cardiometabolic risk. Front. Genet..

[22] Wen X, Triche EW, Hogan JW, Shenassa ED, Buka SL (2011). Prenatal factors for childhood blood pressure mediated by intrauterine and/or childhood growth?. Pediatrics.

[23] Warembourg C (2019). Early-life environmental exposures and blood pressure in children. J. Am. Coll. Cardiol..

[24] Amaral MS (2020). Modeling pathways from the perinatal factors to the vascular risk phenotype at the end of the second decade of life: Birth cohort, Brazil. Hypertension.

[25] Koletzko B (2009). Lower protein in infant formula is associated with lower weight up to age 2 y: A randomized clinical trial. Am. J. Clin. Nutr..

[26] Gruszfeld D (2016). Association of early protein intake and pre-peritoneal fat at five years of age: Follow-up of a randomized clinical trial. Nutr. Metab. Cardiovasc. Dis..

[27] UIS. International Standard Classification of Education: ISCED 1997. Montreal, Canada. *UIS/TD/06-01., UNESCO Libr. Rep. no. 40* (2006).

[28] De Onis M (2007). Development of a WHO growth reference for school-aged children and adolescents. Bull. World Health Organ..

[29] Flynn JT, Falkner BE (2017). New clinical practice guideline for the management of high blood pressure in children and adolescents. Hypertension.

[30] Geerts CC (2007). Tobacco smoke exposure of pregnant mothers and blood pressure in their newborns: Results from the wheezing illnesses study Leidsche Rijn birth cohort. Hypertension.

[31] Oken E, Huh SY, Taveras EM, Rich-Edwards JW, Gillman MW (2005). Associations of maternal prenatal smoking with child adiposity and blood pressure. Obes. Res..

[32] Law CM (1991). Maternal and fetal influences pressure. Arch. Dis. Childhood.

[33] Cabral M (2018). Maternal smoking: A life course blood pressure determinant?. Nicotine Tob. Res..

[34] Lawlor DA (2004). Associations of parental, birth, and early life characteristics with systolic blood pressure at 5 years of age findings from the Mater-University study of pregnancy and its outcomes. Circulation.

[35] Nordenstam F, Norman M, Wickström R (2019). Blood pressure and heart rate variability in preschool children exposed to smokeless tobacco in fetal life. J. Am. Heart Assoc..

[36] Blake KV (2000). Maternal cigarette smoking during pregnancy, low birth weight and subsequent blood pressure in early childhood. Early Hum. Dev..

[37] Morley R, Leeson Payne C, Lister G, Lucas A (1995). Maternal smoking and blood pressure in 7.5 to 8 year old offspring. Arch. Dis. Child..

[38] Bergel E, Haelterman E, Belizan J, Villar J, Carroli G (2000). Perinatal factors associated with blood pressure during childhood. Am. J. Epidemiol..

[39] Roberts RJ (2005). Maternal age in pregnancy and offspring blood pressure in childhood in the Avon Longitudinal Study of Parents and Children (ALSPAC). J. Hum. Hypertens..

[40] Brion MJA, Leary SD, Smith GD, Ness AR (2007). Similar associations of parental prenatal smoking suggest child blood pressure is not influenced by intrauterine effects. Hypertension.

[41] Williams S, Poulton R (1999). Twins and maternal smoking: Ordeals for the fetal origins hypothesis? A cohort study. Br. Med. J..

[42] Dior UP (2014). Parental smoking during pregnancy and offspring cardio-metabolic risk factors at ages 17 and 32. Atherosclerosis.

[43] Högberg L (2012). Effects of maternal smoking during pregnancy on offspring blood pressure in late adolescence. J. Hypertens..

[44] Mamun AA, O’Callaghan MJ, Williams GM, Najman JM (2012). Maternal smoking during pregnancy predicts adult offspring cardiovascular risk factors—evidence from a community-based large birth cohort study. PLoS ONE.

[45] Power C, Atherton K, Thomas C (2010). Maternal smoking in pregnancy, adult adiposity and other risk factors for cardiovascular disease. Atherosclerosis.

[46] Horta BL (2011). Maternal smoking during pregnancy and risk factors for cardiovascular disease in adulthood. Atherosclerosis.

[47] Kataria Y, Gaewsky L, Ellervik C (2019). Prenatal smoking exposure and cardio-metabolic risk factors in adulthood: A general population study and a meta-analysis. Int. J. Obes..

[48] Brion MJA, Leary SD, Lawlor DA, Smith GD, Ness AR (2008). Modifiable maternal exposures and offspring blood pressure: A review of epidemiological studies of maternal age, diet, and smoking. Pediatr. Res..

[49] Vives-Usano M (2020). In utero and childhood exposure to tobacco smoke and multi-layer molecular signatures in children. BMC Med..

[50] Keller G, Zimmer G, Mall G, Ritz E, Amann K (2003). Nephron number in patients with primary hypertension. N. Engl. J. Med..

[51] Al-Odat I (2014). The impact of maternal cigarette smoke exposure in a rodent model on renal development in the offspring. PLoS ONE.

[52] Al-Sawalha NA, AlSari RR, Khabour OF, Alzoubi KH (2019). Influence of prenatal waterpipe tobacco smoke exposure on renal biomarkers in adult offspring rats. Inhal. Toxicol..

[53] Kooijman MN (2015). Fetal smoke exposure and kidney outcomes in school-aged children. Am. J. Kidney Dis..

[54] Totzauer M (2018). Effect of lower versus higher protein content in infant formula through the first year on body composition from 1 to 6 years: Follow-up of a randomized clinical trial. Obesity.

