# Supplementary material for: Effect of maternal smoking during pregnancy on child blood pressure in a European cohort
Source: Sci Rep. 2022 Oct 15;12:17308. doi: 10.1038/s41598-022-21337-7 (PMC9569344; doi:10.1038/s41598-022-21337-7)
Supplement: Supplementary file 1 — Supplementary Tables. [file 41598_2022_21337_MOESM1_ESM.docx]

**Table S1**: Comparison of basic characteristics between the overall population and the analytic sample

|  | **Overall population** | **Included** | **p** |
| --- | --- | --- | --- |
| **N** | 1678 | 572 |  |
| **Sex: female** | 828 (49.3%) | 297 (51.9%) | 0.601 |
| **Country** |  |  | 0.542 |
| Germany | 281 (16.7%) | 80 (14.0%) |  |
| Belgium | 255 (15.2%) | 75 (13.1%) |  |
| Italy | 415 (24.7%) | 145 (25.3%) |  |
| Poland | 275 (16.4%) | 95 (16.6%) |  |
| Spain | 452 (26.9%) | 177 (30.9%) |  |
| **Feeding** |  |  | 0.929 |
| Low protein | 540 (32.2%) | 194 (33.9%) |  |
| High protein | 550 (32.8%) | 185 (32.3%) |  |
| Breastfeeding | 588 (35.0%) | 193 (33.7%) |  |
| **Mother education** |  |  | 0.070 |
| No/Low | 423 (25.3%) | 95 (16.7%) |  |
| Middle | 838 (50.1%) | 292 (51.2%) |  |
| High | 410 (24.5%) | 183 (32.1%) |  |
| **Mother smoked during pregnancy (beyond 12^th^ week)** | 362 (21.6%) | 88 (15.4%) | 0.132 |
| **Birth weight (g): mean (SD)** | 3294 (348) | 3289 (346) | 0.755 |
|  |  |  |  |

**Table S2: Mixed model analysis assessing the effect of MSDP beyond 12^th^ week of gestation on SBP percentile nested by country**

|  | **B (percentile unit) (95% CI)** | | **p-value** |
| --- | --- | --- | --- |
| Intercept | 41.84 | (7.77 – 75.90) | **0.017** |
| Smoked beyond 12th week (yes) | 6.95 | (0.46 – 13.44) | **0.036** |
| Sex (female) | -1.10 | (-5.52 – 3.32) | 0.624 |
| High protein | 3.35 | (-2.07 – 8.77) | 0.225 |
| Breastfeeding | 2.38 | (-3.27 – 8.02) | 0.409 |
| Mother's education level high | 0.13 | (-7.07 – 7.33) | 0.971 |
| Mother's education level middle | 0.84 | (-5.58 – 7.27) | 0.796 |
| Mother's BMI | 0.19 | (-0.38 – 0.75) | 0.524 |
| Father's BMI | 0.24 | (-0.39 – 0.87) | 0.448 |
| Ponderal index at birth | 2.54 | (-7.30 – 12.38) | 0.612 |
| BMI z-score at 11y | 8.30 | (6.34 – 10.26) | **<0.001** |

**Table S3:** Linear regression analysis assessing the effect of smoking beyond 12^th^ week of gestation on diastolic blood pressure percentile at 11 years

|  | **B (percentile unit) (95% CI)** | | **p-value** |
| --- | --- | --- | --- |
| Intercept | -1.766 | (-27.865-24.333) | 0.894 |
| Smoked beyond 12th week (yes) | 2.059 | (-2.833-6.950) | 0.409 |
| Sex | 3.342 | (0.017-6.666) | **0.049** |
| Country: Germany | 29.445 | (23.718- 35.173) | **<0.001** |
| Country: Belgium | 26.199 | (20.718-31.679) | **<0.001** |
| Country: Italy | -2.929 | (-7.484-1.625) | 0.207 |
| Country: Poland | -0.630 | (-6.971-5.711) | 0.845 |
| Low protein | 0.434 | (-3.642-4.511) | 0.834 |
| Breastfeeding | 1.169 | (-2.963-5.301) | 0.579 |
| Mother's education level | 0.184 | (-2.457-2.826) | 0.891 |
| Mother's BMI | 0.252 | (-0.177-.680) | 0.249 |
| Father's BMI | 0.449 | (-0.025-0.923) | 0.063 |
| Ponderal index at birth | 5.551 | (-1.881-12.984) | 0.143 |
| BMI z-score at 11y | 1.665 | (0.188-3.142) | 0.027 |

Sample size 542 patients because of missing data.

Comparison group for country: Spain, and for feeding: high protein

**Table S4:** Linear regression analysis assessing the effect of total number of cigarettes smoked during pregnancy on SBP percentile at 11 years

|  | **B (percentile unit) (95% CI)** | | **p-value** |
| --- | --- | --- | --- |
| Intercept | 57.154 | (-18.485-132.792) | 0.137 |
| Smoked beyond 12th week (yes) | 14.732 | (1.668-27.797) | **0.027** |
| Sex | 0.321 | (-8.799-9.440) | 0.945 |
| Country: Germany | 27.177 | (10.248-44.107) | **0.002** |
| Country: Belgium | -9.721 | (-28.951-9.508) | 0.319 |
| Country: Italy | -1.722 | (-14.995-11.552) | 0.798 |
| Country: Poland | -5.042 | (-23.118-13.034) | 0.582 |
| Low protein | -11.595 | (-21.813- -1.376) | **0.026** |
| Breastfeeding | 0.655 | (-13.740-15.049) | 0.928 |
| Mother's education level | -0.851 | (-8.186-6.485) | 0.819 |
| Mother's BMI | -0.156 | (-1.513-1.201) | 0.821 |
| Father's BMI | 0.505 | (-0.850-1.860) | 0.463 |
| Ponderal index at birth | -1.989 | (-24.944-20.966) | 0.864 |
| BMI z-score at 11y | 8.993 | (4.926-13.060) | **<0.001** |
| Total cigarettes smoked during the pregnancy | -0.003 | (-.007-0.002) | 0.290 |

Sample size 144 patients (children’s no exposed to MSDP excluded)

Comparison group for country: Spain, and for feeding: high protein

**Table S5:** Multiple regression analysis assessing the effect of number of cigarettes/day smoked further 12^th^ week of gestation on SBP percentile at 11 years

|  | **B (percentile unit) (95% CI)** | | **p-value** |
| --- | --- | --- | --- |
| Intercept | 81.680 | (-19.669-183.030) | 0.112 |
| Sex | -1.832 | (-15.238-11.574) | 0.786 |
| Country: Germany | 17.823 | (-6.899- 42.544) | 0.155 |
| Country: Belgium | -11.285 | (-32.035-9.465) | 0.282 |
| Country: Italy | -2.443 | (-21.300-16.414) | 0.797 |
| Country: Poland | -3.619 | (-31.035-23.798) | 0.793 |
| Low protein | -12.707 | (-26.639-1.225) | 0.073 |
| Breastfeeding | -7.194 | (-31.673-17.285) | 0.560 |
| Mother's education level | 3.643 | (-6.651-13.937) | 0.482 |
| Mother's BMI | -0.003 | (-1.894-1.888) | 0.998 |
| Father's BMI | 0.500 | (-1.165-2.164) | 0.551 |
| Ponderal index at birth | -9.860 | (-41.57-721.858) | 0.537 |
| BMI z-score at 11y | 9.928 | (4.482-15.375) | **<0.001** |
| Number of cigarettes/day smoked further 12^th^ week | -0.305 | (-1.960-1.351) | 0.715 |

Sample size 82 (children no exposed to MSDP further 12^th^ week of pregnancy excluded)

Comparison group for country: Spain, and for feeding: high protein
